# Skeletal muscle myostatin mRNA expression is upregulated in aged human adults with excess adiposity but is not associated with insulin resistance and ageing

**DOI:** 10.1007/s11357-023-00956-6

**Published:** 2023-10-06

**Authors:** Andrew Wilhelmsen, Francis B. Stephens, Andrew J. Bennett, Leonidas G. Karagounis, Simon W. Jones, Kostas Tsintzas

**Affiliations:** 1 MRC Versus Arthritis Centre for Musculoskeletal Ageing Research, School of Life Sciences, University of Nottingham, Queen’s Medical Centre, Nottingham, NG7 2UH UK; 2 Sport and Health Sciences, University of Exeter, Exeter, UK; 3 School of Life Sciences, University of Nottingham, Queen’s Medical Centre, Nottingham, UK; 4 Mary MacKillop Institute for Health Research (MMIHR), Melbourne, Australian Catholic University, Melbourne, Australia; 5 Institute of Social and Preventive Medicine (ISPM), University of Bern, Bern, Switzerland; 6 Institute of Inflammation and Ageing, MRC Versus Arthritis Centre for Musculoskeletal Ageing Research, Queen Elizabeth Hospital, The University of Birmingham, Birmingham, UK

**Keywords:** Myostatin, Insulin resistance, Skeletal muscle, Primary human myotubes, Obesity, Ageing

## Abstract

Myostatin negatively regulates skeletal muscle growth and appears upregulated in human obesity and associated with insulin resistance. However, observations are confounded by ageing, and the mechanisms responsible are unknown. The aim of this study was to delineate between the effects of excess adiposity, insulin resistance and ageing on myostatin mRNA expression in human skeletal muscle and to investigate causative factors using in vitro models. An in vivo cross-sectional analysis of human skeletal muscle was undertaken to isolate effects of excess adiposity and ageing per se on myostatin expression. In vitro studies employed human primary myotubes to investigate the potential involvement of cross-talk between subcutaneous adipose tissue (SAT) and skeletal muscle, and lipid-induced insulin resistance. Skeletal muscle myostatin mRNA expression was greater in aged adults with excess adiposity than age-matched adults with normal adiposity (2.0-fold higher; *P* < 0.05) and occurred concurrently with altered expression of genes involved in the maintenance of muscle mass but did not differ between younger and aged adults with normal adiposity. Neither chronic exposure to obese SAT secretome nor acute elevation of fatty acid availability (which induced insulin resistance) replicated the obesity-mediated upregulation of myostatin mRNA expression in vitro. In conclusion, skeletal muscle myostatin mRNA expression is uniquely upregulated in aged adults with excess adiposity and insulin resistance but not by ageing alone. This does not appear to be mediated by the SAT secretome or by lipid-induced insulin resistance. Thus, factors intrinsic to skeletal muscle may be responsible for the obesity-mediated upregulation of myostatin, and future work to establish causality is required.

## Introduction

Obesity is typically associated with declining metabolic health, including impaired regulation of glucose homeostasis and metabolism through the action of insulin (insulin resistance). Often concurrent with insulin resistance is an impairment of the amino acid stimulation of muscle protein synthesis, termed anabolic resistance [1, 2]. Together, the obesity-associated impairment of insulin and anabolic sensitivity contribute to pathophysiological consequences with ageing, including the decline of skeletal muscle mass and function, termed sarcopenia [3, 4]. Since skeletal muscle constitutes the greatest mass of the insulin-sensitive tissues, with a capacity to increase glucose disposal many-fold from post-absorptive to postprandial states, the diminution of skeletal muscle mass directly reduces whole body glucose disposal and jeopardizes whole-body glucose homeostasis [5]. Therefore, the implications of ageing and obesity on factors involved in the regulation of skeletal muscle mass are entwined with those of glucose and energy homeostasis.

Myostatin is a TGF-β family member that is predominantly expressed and secreted from skeletal muscle, functioning as a negative regulator of skeletal muscle growth [6]. As outlined in a recent review [7], myostatin acts through both genomic and non-genomic signalling events to affect skeletal muscle mass by increasing the expression of proteolytic genes, decreasing the expression of muscle-specific transcription factors and structural genes, and reducing anabolic signalling (such as mTORC1 inhibition) in myofibres. Moreover, studies in mice indicate that long-term myostatin inhibition does not operate through satellite cells to affect myofibre hypertrophy [8–10]. Following observations of resistance to high-fat diet-induced impairment of glucose tolerance in myostatin-knockout mice [11], it was reported in humans that greater myostatin expression was associated with insulin resistance [12, 13]. Concordantly, the skeletal muscle mRNA and protein expression and circulating abundance of myostatin are reportedly elevated with obesity and correlate with body mass index (BMI) [12, 14, 15]. Indeed, individuals with greater skeletal muscle myostatin expression were found to require lower rates of glucose infusion during euglycaemic-hyperinsulinaemic clamp to maintain normoglycaemia [16].

Potential causality of the relationship between elevated myostatin and insulin resistance has been demonstrated in mice and murine culture models, where treatment with recombinant myostatin perturbed indices of insulin sensitivity and signalling, as well as glucose tolerance [17, 18], whereas inhibition of myostatin has been associated with improved insulin sensitivity [19–21]. To date, cross-sectional studies in humans have predominantly investigated myostatin expression in middle-aged and aged individuals with and without obesity. It must be considered, however, that adiposity, insulin resistance and ageing often advance together [22].

In the absence of obesity, some studies have reported elevated myostatin serum abundance and muscle mRNA and protein expression in aged adults when compared with younger adults [23–25], but firm conclusions are limited by the absence of BMI-matched younger and aged adults and by differences in body composition [26, 27]. Thus, it remains unclear whether changes in the expression and abundance of myostatin are driven by excess adiposity per se or are influenced by age-related changes such as development of insulin resistance and declining skeletal muscle mass and function [28, 29]. Furthermore, the causative factors responsible for the reported upregulation of myostatin with obesity and ageing remain unclear.

## Aims

This study sought to delineate between the effects of excess adiposity, insulin resistance and ageing on myostatin mRNA expression in human skeletal muscle and to undertake in vitro investigations using human primary muscle cultures to explore possible causative factors.

## Materials and methods

### In vivo procedures and human tissue acquisition

All in vivo procedures were performed following approval from the Medical School research ethics committee of the University of Nottingham and in accordance with the declaration of Helsinki. Volunteers provided written informed consent prior to participating and underwent a comprehensive medical screening.

### Cross-sectional analysis of ageing and obesity

A cross-sectional analysis of skeletal muscle mRNA expression in human ageing and obesity was undertaken. Healthy male volunteers were recruited who were either young with normal adiposity (YNA; 18–30 years, mean [± *SD*] *BMI*: 23.8 [3.8] kg·m^−2^), aged with normal adiposity (ANA; ≥ 65 years, *BMI*: 23.6 [2.5] kg·m^−2^) or aged with excess adiposity (AEA; ≥ 65 years, *BMI*: 29.6 [2.1] kg·m^−2^). Individuals were excluded if they had a history of diabetes, cardiovascular disease, hypertension, or any other metabolic or respiratory conditions. Following an overnight fast and 48 h of abstinence from strenuous physical activity, a dual energy X-ray absorptiometry (DEXA) scan (Lunar Prodigy, GE Healthcare, USA) was undertaken to determine body composition before a skeletal muscle tissue sample was obtained from the vastus lateralis using the suction-modified Bergström biopsy technique and snap frozen [30]. A retrograde cannula was inserted into a superficial hand vein, which was kept in a warming unit (55 °C) to enable arterialized-venous sampling. Whole body insulin sensitivity was assessed using a 3-h hyperinsulinaemic-euglycaemic clamp, as previously described [31–33]. Insulin was continuously infused at 60 mU·m^−2^·min^−1^, and 20% glucose was infused at a variable rate to achieve euglycaemia (set at 4.5 mmol·L^−1^). Thus, glucose infusion rate was used to calculate the rate of glucose disposal, which was adjusted for total lean mass (LM) calculated from the DEXA scan and expressed as μmol·kg^−1^ LM·min^−1^.

### Blood biochemistry

Whole blood glucose concentration was determined using the glucose oxidase method (YSI 2300 STAT PLUS Glucose & Lactate Analyzer, Yellow Springs Instruments, Ohio, USA). Serum insulin was measured with a commercially available enzyme-linked immunosorbent assay (EZHI-14K, Millipore Sigma). The homeostasis model assessment of insulin resistance 2 (HOMA2-IR) was used as an index of fasting insulin sensitivity [34].

### Acquisition of subcutaneous adipose tissue for modelling adipose-muscle crosstalk

For the purpose of modelling adipose-muscle crosstalk in subsequent in vitro experiments, subcutaneous adipose tissue (SAT) samples were obtained from adults (range: 48–84 years) with normal adiposity (*n* = 18; *mean* [± *SD*] *BMI*: 24.2 [1.4] kg·m^−2^) or excess adiposity (*n* = 15; *BMI*: 32.8 [3.2] kg·m^−2^), who had knee or hip osteoarthritis (OA) and were undergoing orthopaedic surgery at the Royal Orthopaedic Hospital, Birmingham (NRES 16/SS/0172). Patients who had ever received intravenous or oral immunosuppressive medication and those who had been given intra-articular steroid injections within 6 months of their surgery were all excluded.

### In vitro procedures

#### **Generation and differentiation of primary human myogenic cultures**

For in vitro experiments, a total of *n* = 13 healthy participants attended the laboratory on a single occasion, following an overnight fast and 48-h abstinence from strenuous physical activity. A venous blood sample was taken for determination of fasting glucose concentration before a sample of vastus lateralis muscle was obtained via the suction-modified Bergström biopsy technique and briefly stored in ice-cold saline prior to the isolation of progenitor cells. Primary myogenic cultures were generated and differentiated (for 6 days) to form multinucleated myotubes, as previously described [35]. Based on unpublished findings, cultures were only passaged twice, to maintain myogenic purity and phenotype.

### Modelling subcutaneous adipose tissue cross-talk with skeletal muscle

To generate adipose-conditioned medium (ACM), SAT samples were incubated in differentiation medium at a ratio of 1 g of tissue to 10 mL of medium for 24 h. Larger samples were divided into segments of ~1 g to standardize the surface area of adipose tissue. The ACM was sterile filtered and stored at −80 °C. Prior to use, ACM from numerous patients (normal adiposity: *n* = 18; excess adiposity: *n* = 15) was combined to generate a stock of normal adiposity and a stock of excess adiposity ACM, which were matched for mean age and sex distribution (Table 2). The relative abundance of 58 adipokines within both pooled ACM stocks was assessed using a commercially available human adipokine array kit (R&D Systems, #ARY204).

Upon reaching > 90% confluence, myogenic cultures from *n* = 4 healthy participants (*BMI* range: 19.8–24.5 kg·m^−2^) were switched to either unconditioned, normal adiposity ACM or excess adiposity ACM for differentiation. Freshly-thawed ACM was diluted 1:2 with fresh differentiation medium and was renewed every 48 h for 6 days [36].

#### Induction of lipid-induced insulin resistance

Primary myogenic cultures were established from *n* = 8 healthy, normoglycaemic (fasting blood glucose < 5.0 mmol·L^−1^) participants (*BMI* range: 20.3–26.9 kg·m^−2^). Palmitate, linoleic and oleic acids were coupled to fatty acid-free BSA at molar ratios of 1:2.7. Fully differentiated myotubes were exposed for 16 h with either palmitate (250 μM) alone, a mixture of palmitate, linoleic acid and oleic acid (PLO; 250, 50 and 150 μM, respectively) or vehicle (fatty acid-free BSA).

#### Glucose uptake

Basal and insulin-stimulated (100 nM) glucose uptake was assessed in myotubes using nominally radio-labelled (tritiated) deoxy-D-glucose (2-[1,2-3H (N)]; PerkinElmer, MA, USA), as previously described [37]. Additionally, aliquots of cell lysate were assayed for protein concentration determination by BCA assay; the remaining lysates were combined with scintillation fluid, and beta-particle emission (disintegrations per minute; DPM) was assessed via liquid scintillation counting. DPMs were normalized against protein content and converted to absolute glucose uptake rate (pmol·mg^−1^·min^−1^).

#### Myotube diameter

Differentiated myogenic cultures were fixed with 4% paraformaldehyde for 15 min before permeabilization with 0.3% Triton X-100 for 20 min. Cells were blocked for 2 h in 5% (w/v) BSA with 0.1% Triton X-100 and incubated in anti-desmin antibody (Abcam, #ab8592) in 1% BSA with 0.1% Triton X-100 overnight. Cells were then incubated in secondary antibody (Invitrogen, #R37118) in 1% BSA with 0.1% Triton X-100 for 1 h before 1 μg·mL^−1^ DAPI (Sigma, #32670) was added. Cultures were imaged on a fluorescent microscope platform (Evos M7000, Thermo Fisher). Ten images were captured from random locations across each well, with 3 replicate wells per experimental treatment. Image analysis was performed in ImageJ. For each image, the diameter of the 5 largest desmin-positive multinucleated tubular structures was measured in 3 places along their length at 25, 50 and 75% of the length of the visible structure, to calculate a mean diameter for each myotube. Thus, myotube diameter (μm) data for each experimental treatment reflects the mean of 150 unique myotubes.

#### Global protein synthesis

Changes in amino acid stimulated global myotube protein synthesis were estimated using the surface sensing of translation (SuNSET) technique [38]. Fully differentiated myotubes were serum-starved for 2 h and subsequently incubated in a transport buffer (140 mM NaCl, 1.8 mM CaCl_2_, 0.08 mM MgSO_4_, 5.4 mM KCl, 25 mM HEPES and pH 7.4) supplemented with 5 mM D-glucose and 2 mM L-leucine for 3 h to stimulate protein synthesis. For the final 30 min, cells were incubated with 1 μM puromycin dihydrochloride before lysis. Equal amounts of total protein (determined via Pierce bicinchoninic acid assay) were separated on 12% SDS-PAGE gels, transferred onto PVDF membranes, incubated with anti-puromycin antibody (clone 12D10; Millipore, USA) and visualized using chemiluminescence on X-ray film. Following immunodetection, membranes were stained with 0.1% Coomassie. Films and membranes were digitally imaged and total lane intensity (~15–200 kDa) was measured by densitometry. Relative protein synthetic activity was estimated as the ratio of puromycin-labelled protein intensity to total protein (Coomassie) intensity.

#### mRNA expression

Extraction of RNA was performed on both skeletal muscle tissue and cultured cells using TRIzol™ (Thermo Fisher), whilst cDNA synthesis was performed using Invitrogen SuperScript™ in accordance with manufacturer’s guidelines. Relative gene expression was assessed via real time PCR (qPCR) with TaqMan probes (custom designed or commercially available assay kits) on a StepOnePlus™ system (Applied Biosystems, CA, USA). Expression of target and endogenous reference genes were determined using the relative standard curve method [39]. The stability of candidate endogenous reference genes was assessed using NormFinder [40].

#### Terminology to describe biological and technical replication

For in vitro experiments, an independent donor repeat refers to the performing of an experiment using primary myogenic cultures established from a single human donor (e.g. the use of *n* = 7 independent donor repeats meant the entire experimental model was repeated 7 times using cultures established from 7 different human donors). Within each independent donor repeat, multiple treatment replicates were performed (e.g. the use of 4 treatment replicates meant 4 wells of cells from each independent donor repeat were exposed to each treatment condition). All reported *n* values are the number of independent donor repeats.

### Statistical analysis

All statistical analysis was performed in GraphPad Prism (Version 9.2). Descriptive data are presented as *mean* ± *SD*, whilst experimental data are presented as *mean* ± *SEM*. Assumptions of normality were tested via Shapiro-Wilk test. Analysis of variance (ANOVA) was applied for complete data sets; for data with missing repeated measures (due to insufficient primary cells to repeat all experimental conditions within an independent donor repeat), mixed-effects models were employed. Where relevant, ANOVA/mixed effects model *P* values are reported, with post hoc Tukey multiple comparisons. Standalone comparisons were performed with paired two-sided *t*-tests. Correlations were performed using Pearson’s correlation. Statistical significance was defined as *P* < 0.05. All individual participant data points for in vivo experimental outcomes and all independent donor repeat data points for in vitro experimental outcomes are presented as circles on each figure, with the number of in vitro treatment replicates reported in the legend, where relevant.

## Results

### In vivo studies

1. Skeletal muscle mRNA expression of myostatin is increased and insulin sensitivity is impaired in aged adults with excess adiposity, but is unaffected by ageing alone.
  Participants in the ANA and AEA groups were age-matched and were on average ~50 years older than those in the YNA group (Table 1). The AEA group presented with higher *BMI*, total fat mass and body fat percentage than both ANA and YNA. Insulin-stimulated glucose disposal (an index of insulin sensitivity) was not significantly different between YNA and ANA but was significantly lower in AEA than both ANA and YNA (Table 1), suggesting that increased adiposity with ageing, but not ageing alone, impairs insulin sensitivity in otherwise healthy individuals.

**Table 1:** Characteristics of participants from whom skeletal muscle samples were obtained in the in vivo studies

	Young normal adiposity (YNA)	Aged normal adiposity (ANA)	Aged excess adiposity (AEA)	*P*
*n*	6	7	7	
Age (years)	22.4 (± 3.7)	70.6 (± 1.1)†	69.1 (± 1.1)†	**< 0.001**
Body mass (kg)	77.8 (± 15.0)	69.7 (± 9.2)	85.3(± 6.8)‡	**0.036**
*BMI* (kg·m^−2^)	23.8 (± 3.8)	23.6 (± 2.5)	29.6 (± 2.1)†‡	**0.002**
Total lean mass (LM) (kg)	58.92 (± 8.26)	50.98 (± 3.92)	52.82 (± 4.80)	0.063
Total fat mass (kg)	13.36 (± 7.98)	14.42 (± 5.97)	27.13 (± 3.03)†‡	**< 0.001**
Body fat percentage (%)	16.3 (± 7.2)	20.1 (± 6.6)	31.8 (± 2.0)†‡	**< 0.001**
Glucose disposal rate (μmol·kg^−1^ LM·min^−1^)	74.2 (± 16.0) (*n* = 5)	57.2 (± 6.5) (*n* = 5)	41.9 (± 12.9)†‡ (*n* = 7)	**0.003**

Data are presented as mean ± *SD*

†Significantly different from young normal adiposity

**Fig. 1:**
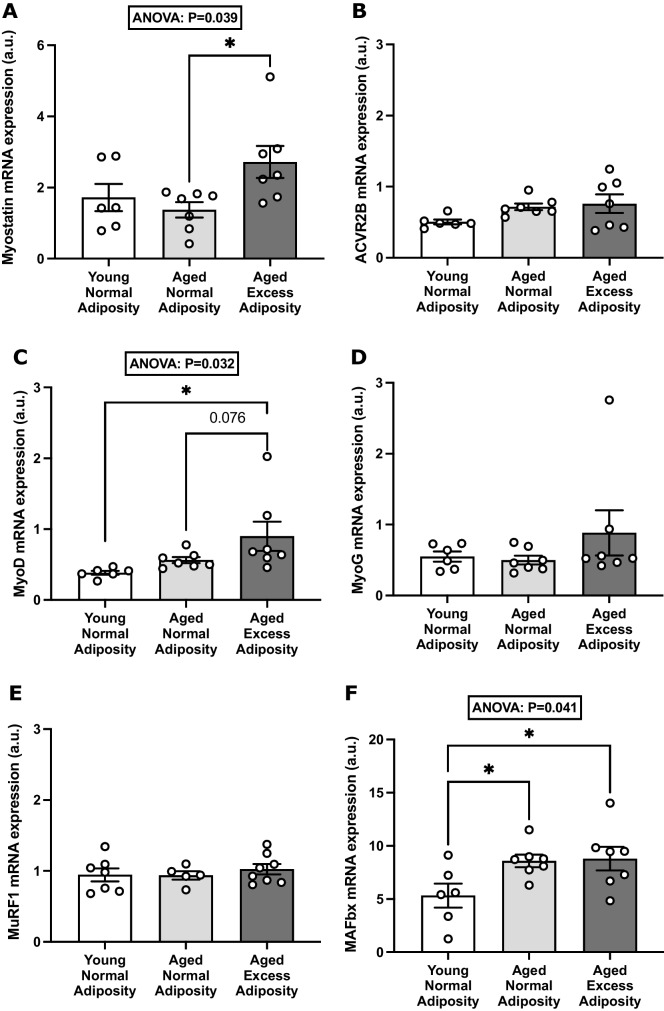
Effects of adiposity and ageing on myostatin and muscle regulatory gene expression in skeletal muscle tissue. The relative mRNA expression of myostatin (**A**), ACVR2B (**B**), MyoD (**C**), MyoG (**D**), MuRF1 (**E**) and MAFbx (**F**) was measured via qPCR in vastus lateralis muscle samples from adults who were young with normal adiposity (*n* = 6), aged with normal adiposity (*n* = 7) and aged with excess adiposity (*n* = 7). Group data are presented as *mean* ± *SEM*, with individual participant values presented as open circles. Relative mRNA expression is normalized against the geometric mean of HMBS and RPLP0 (a.u.). * *P* < 0.05
  In order to better contextualize the differences in myostatin mRNA expression observed between the YNA, ANA and AEA groups, we next investigated whether these differences extended to the expression of key muscle-regulatory genes. To this end, the mRNA expression of the myogenic factors MyoD and MyoG and the catabolic muscle-specific E3 ligases MAFbx and MuRF1 were quantified in the same skeletal muscle samples. The mRNA expression of MyoD was 2.3-fold greater in AEA than YNA (mean difference [95% *CI*]: 0.52 [0.04, 0.99] a.u.; *P* < 0.05) and tended (*P* = 0.076) to be greater than ANA (Fig. 1C). Conversely, expression of MyoG was not significantly affected by experimental group (Fig. 1D). There was also no effect of group on the expression of MuRF1 (Fig. 1E). However, MAFbx expression was significantly affected by group (*P* = 0.041), with ~1.6-fold greater expression in AEA (mean difference [95% *CI*]: 3.48 [0.56, 6.40] a.u.; *P* < 0.05) and ANA (mean difference [95% *CI*]: 3.26 [0.35, 6.18] a.u.; *P* < 0.05) than YNA (Fig. 1F).

‡Significantly different from aged normal adiposity
  Skeletal muscle mRNA expression of myostatin was 2.0-fold greater in AEA than ANA (mean difference [95% *CI*]: 1.35 [−0.06, 2.63] a.u.; *P* < 0.05), but no difference was observed between ANA and YNA (Fig. 1A). Across all participants, myostatin mRNA expression tended to weakly correlate with *BMI* (*r* = 0.38; *P* = 0.097; data not shown), but not with any other indices of body composition (body mass, total fat mass, trunk fat mass, body fat % and lean mass), or with age or glucose disposal rate. Furthermore, no significant differences between groups in the skeletal muscle mRNA expression of the myostatin receptor, ACVR2B, were observed (Fig. 1B). However, when only the aged individuals (AEA and ANA groups) were considered, myostatin expression showed a significant positive correlation with *BMI* (*r* = 0.65, *P* = 0.011; *n* = 14) and a negative correlation with lean mass when expressed as % of body mass (*r* = −0.61, *P* = 0.021; *n* = 14).
2. Aged adults with excess adiposity exhibit altered expression of key skeletal muscle-regulatory genes, relative to aged and younger counterparts with normal adiposity.

### In vitro studies

**Fig. 2:**
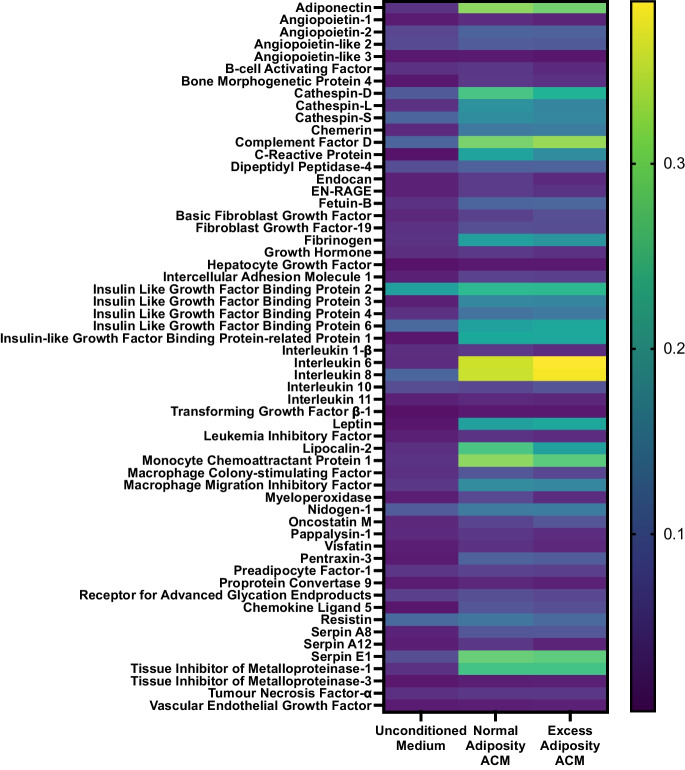
Relative abundances of adipose-secreted cytokines in unconditioned medium and ACM from adults with normal or excess adiposity. The relative abundance of 58 cytokines known to be secreted from adipose tissue was measured in the pooled stocks of ACM from adults with normal adiposity (*n* = 18) and excess adiposity (overweight/obese; *n* = 15) using a commercially available human adipokine array kit. Lower relative abundance is indicated by darker, bluer shades, whilst higher abundance is indicated by brighter, yellower shades

**Fig. 3:**
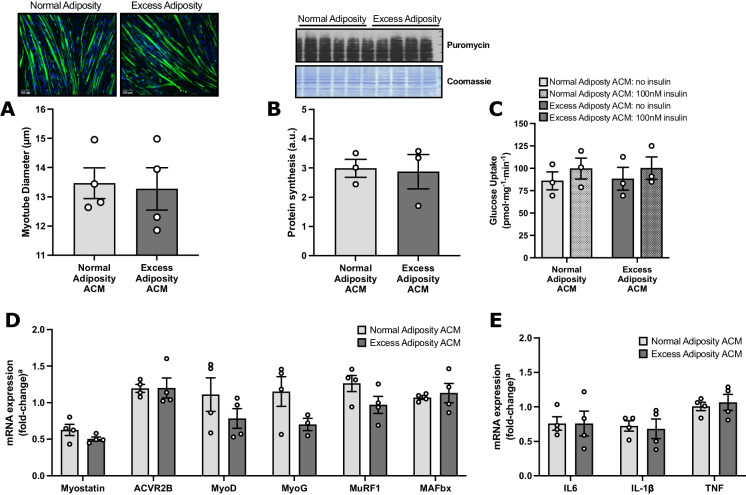
Effects of chronic exposure to subcutaneous ACM from adults with excess adiposity on factors associated with the regulation of skeletal muscle mass, insulin sensitivity and inflammation in primary human myotubes. Primary human myoblasts were exposed to subcutaneous ACM from adults with normal adiposity or excess adiposity for 6 days of differentiation into myotubes. Mean myotube diameter (*n* = 4; **A**), with representative micrographs of fluorescently stained myotubes (desmin (green); DAPI (blue); 10× objective; scale bar = 100 μm). Amino acid stimulated global myotube protein synthesis as indicated by the relative incorporation of puromycin into newly synthesized peptides (*n* = 3; **B**). Basal and insulin-stimulated radio-labelled 2-DOG uptake (*n* = 3; **C**). Relative mRNA expression of genes involved in skeletal muscle mass regulation (*n* = 4; **D**) and inflammation (*n* = 4; **E**). Data are presented as *mean* ± *SEM*, with independent donor repeats presented as open circles; each independent donor repeat represents the mean of 4–6 treatment replicates. ^a^Relative mRNA expression is expressed as fold-change from cells treated with unconditioned medium

**Fig. 4:**
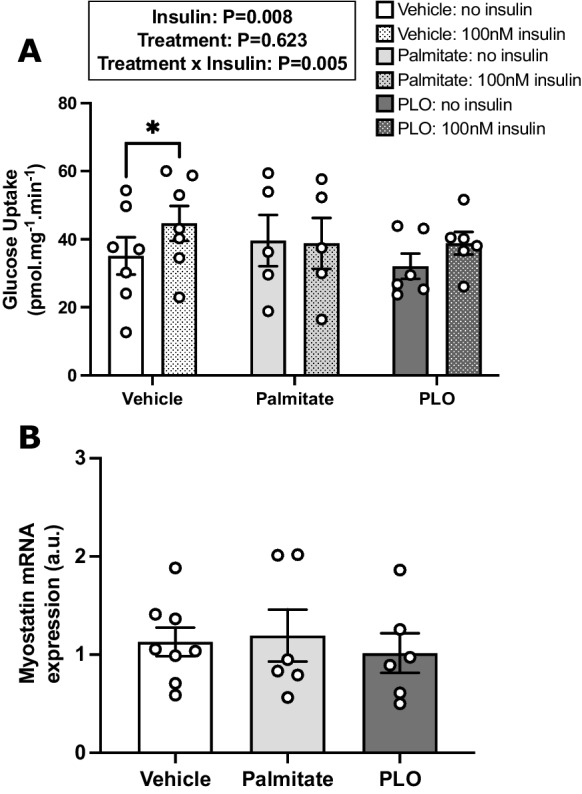
Effects of acute fatty acid treatments on glucose uptake and myostatin gene expression in primary human myotubes. Basal (no insulin) and insulin-stimulated radio-labelled 2-DOG uptake (**A**) were assessed following 16-h treatment with vehicle (*n* = 7), palmitate (*n* = 5) or PLO (*n* = 6). Relative mRNA expression of myostatin (**B**) following vehicle palmitate (*n* = 6) and PLO (*n* = 6) treatment, expressed as fold-change from vehicle (*n* = 8). Data are presented as *mean* ± *SEM*, with independent donor repeat mean values presented as open circles; each independent donor repeat represents the mean of 4–6 (**A**) and 3–4 (**B**) treatment replicates. * *P* < 0.05 from no insulin treatment

1. Subcutaneous adipose tissue secretome from aged adults with excess adiposity does not differentially modify insulin sensitivity and myostatin mRNA expression in human myotubes, compared to that from individuals with normal adiposity.
  Given the observed increase in skeletal muscle myostatin mRNA expression with adiposity in the presence of ageing, in vitro studies were conducted to investigate potential causative drivers. In the first instance, experiments investigated whether cross-talk between SAT and skeletal muscle might play a causal role in the obesity-mediated upregulation of myostatin. The patients with normal adiposity (*n* = 18) and excess adiposity (*n* = 15) from whom SAT samples were obtained for the generation of ACM were characteristically different (Table 2). Compared to ACM derived from patients with normal and excess adiposity, unconditioned differentiation medium demonstrated vastly lower abundances of most measured cytokines, confirming successful conditioning with SAT secretome (Fig. 2). The relative abundances of several of the 58 cytokines assessed demonstrated anticipated differences between the normal adiposity and excess adiposity ACM. Notably, compared to ACM from patients with normal adiposity, ACM from patients with excess adiposity demonstrated a 6% lower relative abundance of adiponectin, whilst interleukins 6, 8 and 10 were all more abundant (9%, 9% and 27%, respectively).
  Exposure to SAT secretome from patients with excess adiposity did not impair myotube formation, with comparable myotube diameters in cells treated with normal adiposity and excess adiposity ACM (13.46 ± 0.53 vs. 13.27 ± 0.73 μm, respectively; *n* = 4; Fig. 3A). Both amino acid-stimulated global myotube protein synthesis (estimated via puromycin incorporation; *n* = 3) and basal and insulin-stimulated glucose uptake (*n* = 3) were comparable between ACM treatments (Fig. 3 B and C, respectively). The relative mRNA expression of myostatin and its receptor, ACVR2B, as well as the relative expression of MRFs and muscle-specific E3 ligases were similarly comparable between ACM treatments (all *n* = 4; Fig. 3D). Relative to cells treated with normal adiposity ACM, cells treated with excess adiposity ACM did not demonstrate altered expression of IL-6, IL-1β or TNF (Fig. 3).
2. Fatty acid mediated impairment of human myotube insulin sensitivity is not associated with changes in the mRNA expression of myostatin.
  Given that skeletal muscle mRNA expression of myostatin was increased and insulin sensitivity was impaired in participants with excess adiposity in our in vivo study, we next sought to examine whether the induction of insulin resistance can directly affect myostatin expression. To this end, primary myogenic cultures were established from *n* = 8 young healthy donors (23 ± 3 years, *BMI*: 23.1 ± 2.6 kg·m^−2^) with normal insulin sensitivity (HOMA2-IR: 1.12 ± 0.38). In fully differentiated myotubes, basal glucose uptake was unaffected by fatty acid treatment (*n* = 7); however, under insulin-stimulated conditions, treatment with both palmitate (*n* = 5) and PLO (*n* = 6) impaired the significant increase in glucose uptake observed in the vehicle-treated myotubes (vehicle: *mean ± SEM* fold-change from basal to insulin-stimulated: 1.4 ± 0.25; mean difference [95% *CI*]: 9.5 [18.0, 1.1] pmol·mg^−1^·min^−1^; *P* < 0.05), indicating the development of insulin resistance in both palmitate- and PLO-treated cells (Fig. 4A). However, there was no effect of fatty acid treatment on myostatin mRNA expression (*n* = 8; Fig. 4B), suggesting that the insulin resistance observed in aged adults with excess adiposity in the in vivo study may not be functionally linked to upregulation of skeletal muscle mRNA expression of myostatin.

**Table 2:** Characteristics of participants from whom subcutaneous adipose tissue samples were obtained for the generation of adipose-conditioned medium

	Normal adiposity	Excess adiposity	*P*
Sex (*n*: M/F)	7/11	5/10	-
Age (years)	70 (± 10)	68 (± 10)	0.587
Height (m)	1.65 (± 0.09)	1.66 (± 0.08)	0.836
Weight (kg)	66.3 (± 8.1)	90.5 (± 12.8)	**< 0.001**
*BMI* (kg·m^−2^)	24.2 (± 1.4)	32.8 (± 3.2)	**< 0.001**
Waist circumference (cm)	88.0 (± 9.5)	104.0 (± 10.9)	**< 0.001**
Hip circumference (cm)	99.7 (± 7.9)	116.9 (± 8.0)	**< 0.001**

Data are presented as mean ± *SD*

## Discussion

This is the first study to demonstrate that whilst myostatin mRNA expression is upregulated in the skeletal muscle of aged adults with excess adiposity who are insulin resistant, this upregulation is independent of chronological ageing. Furthermore, the in vitro models used in the present study suggest that this upregulation is not mediated by the induction of insulin resistance or by cross-talk with the subcutaneous adipose tissue secretome in the setting of excess adiposity. Thus, we provide novel evidence suggesting a dissociation between skeletal muscle insulin resistance and the upregulation of myostatin mRNA expression in human obesity.

Aged adults with excess adiposity, who were also insulin resistant, demonstrated an almost 2-fold higher skeletal muscle expression of myostatin mRNA relative to age-matched aged adults with normal adiposity. This occurred without a concomitant change in the mRNA expression of the transmembrane receptor, ACVR2B, to which myostatin binds. The finding of upregulated muscle myostatin expression in the presence of excess adiposity and the correlation between myostatin expression and *BMI* is consistent with previous reports [12, 13, 15]. The novel finding of this study is that in the absence of excessive adiposity, ageing alone was not associated with a change in myostatin gene expression in human skeletal muscle. Whether such upregulation with adiposity translates into elevated circulating myostatin protein, however, is less clear [41–43]. Furthermore, it is not known to what extent this upregulation in muscle myostatin mRNA expression in aged adults with excess adiposity is causally related to their lower lean mass when expressed as percent of body mass when compared to those with normal adiposity.

The mechanism by which myostatin is upregulated in obesity has not yet been fully established; however, canonical myostatin signalling activates SMAD2/3 which represses muscle regulatory factors (MRFs), including MyoD [44–47]. Conversely, MyoD itself is also able to upregulate myostatin transcription through its binding to enhancer-box motifs within the myostatin promoter region [48–50]. Thus, to contextualize the differences in muscle myostatin mRNA expression, we investigated whether the differences with excess adiposity and ageing extended to key muscle regulatory genes. Relative to the YNA group, the mRNA expression of the MRF, MyoD, was significantly greater in AEA. The capacity for MyoD to upregulate myostatin provides a potential mechanistic link between their concurrent upregulation in the AEA group, whereby the initial upregulation of MyoD in ageing with obesity may precede and promote the upregulation of myostatin. This finding, however, is at odds with reports of diminished MyoD in younger-to-middle-aged adults with obesity [51], possibly suggesting that perturbed regulation of MyoD is a feature only seen in more advanced age, which is consistent with rodent studies of ageing [52, 53].

In the present study, the mRNA expression of the muscle-specific E3 ubiquitin ligase MAFbx, but not MuRF1, was increased in aged participants. Although research into the involvement of the ubiquitin-proteasome system in age-related muscle wasting is conflicting [54–57], it has been demonstrated that ex vivo incubation of skeletal muscle with full-length myostatin protein increases muscle proteolysis mediated by MuRF1 and MAFbx [58]. Whilst this suggests a means by which upregulated myostatin expression in the presence of greater adiposity could be deleterious to the maintenance of skeletal muscle mass with ageing, further investigation is required to establish whether this is initiated by upregulation at the mRNA level.

Having observed that the upregulation of myostatin mRNA expression with excess adiposity occurs independent of ageing alone, we next investigated possible causative factors. To that effect, the increased abundance and hypertrophy of adipocytes and resident/infiltrating immune cells in obesity promote the elevated secretion and subsequently circulating abundance of inflammatory factors [59–61]. Through this secretory function, excessive adiposity conveys pleiotropic effects on endocrine and metabolic function, contributing to pathophysiological consequences with ageing [62, 63]. Thus, in vitro experiments were undertaken to isolate the effects of cross-talk between SAT, constituting the largest adipose compartment in obesity, and skeletal muscle on myostatin mRNA expression.

When primary myotubes were differentiated for 6 days in ACM, the mRNA expression of myostatin was not significantly different between cells exposed to the SAT of patients with normal adiposity versus that of patients with excess adiposity. Exposure to the SAT secretome of patients with excess adiposity did not impair basal or insulin-stimulated glucose uptake or amino acid-stimulated global protein synthesis, demonstrating an absence of SAT secretome-induced insulin or anabolic resistance in the setting of excess adiposity. Concordantly, the relative mRNA expression of MRFs, muscle-specific E3 ubiquitin ligases and classical inflammatory genes were similar between treatments, whilst myotube diameter was similarly unaffected, the latter of which is consistent with previous findings in primary human myotubes from young donors [36].

Previously, the secretome of other adipose depots has been used in cross-talk experiments with skeletal muscle cells, with contrasting results to the present study. Pellegrinelli et al. demonstrated that co-culture of myotubes with obese donor visceral adipocytes for 24 h reduced the mRNA expression of MyoD and MyoG, but not MuRF1 or MAFbx and was accompanied by impaired muscle protein synthetic signalling [64]. In another study, exposure of primary human myotubes to mammary ACM derived from normal weight and overweight donors for 48 h downregulated the expression of MyoD, MyoG and myosin heavy chain (MHC), relative to unconditioned medium [65]. Supportively, acute (6 h) exposure of L6 myotubes to visceral, but not SAT secretome from individuals with extreme obesity impaired insulin-stimulated glucose uptake [66, 67]. Together, these findings suggest differential effects of adipose tissues on myogenesis and muscle insulin sensitivity in excess adiposity. Thus, whilst the secretory phenotype of adipose tissue in obesity is broadly associated with deleterious cross-talk with skeletal muscle, under the experimental conditions employed in the current study, exposure to the obese SAT secretome does not explain the upregulated mRNA expression of myostatin observed in vivo. It remains to be investigated whether ectopic fat accumulation, such as intramyocellular lipid content, which is elevated in aged adults with obesity and insulin resistance [68, 69], play a causative role in the upregulation of skeletal muscle myostatin.

In the absence of upregulation in myostatin mRNA expression or induction of insulin resistance with chronic exposure to the SAT secretome of patients with excess adiposity, further in vitro experiments were undertaken to induce overt insulin resistance and to establish whether this is associated with upregulation of myostatin mRNA expression. Indeed, insulin resistance has previously been associated with increased mRNA expression and serum abundance of myostatin in humans [12, 16, 41]. It is well established that acute lipid overloads can induce skeletal muscle insulin resistance under some experimental conditions [70, 71] and this lipid-induced insulin resistance can be modelled in vitro through the incubation of myotubes with excess saturated fatty acids [72]. Using the latter model, the present study demonstrated for the first time in primary human myotubes that the mRNA expression of myostatin was unaffected by lipid-induced insulin resistance. It has previously been demonstrated in animal models that the upregulation or administration of myostatin promotes insulin resistance, which may involve the repression of insulin-stimulated protein kinase B (Akt) phosphorylation by the activation of SMAD2/3 [73], which subsequently impairs the distal insulin signaling cascade [17, 18, 74]. Additionally, myostatin has been shown to repress AMP-activated protein kinase (AMPK) expression and activity [19, 75, 76], which could be detrimental to skeletal muscle glucose handling and insulin sensitivity [77].

Taken together, these findings suggest that whilst the upregulation of myostatin may exacerbate insulin resistance in individuals with excess adiposity, lipid-induced insulin resistance does not induce its upregulation. Thus, neither the induction of insulin resistance nor cross-talk with SAT is able to explain the obesity-mediated upregulation of myostatin in ageing. It is important to also consider, therefore, the protective role of exercise in the regulation of skeletal muscle mass and glucose metabolism in the context of advancing age. Crucially, age-related decline in the structure and function of skeletal muscle can be mitigated through regular exercise via myriad remodelling mechanisms, some of which are age-independent [78]. As evidenced by studies of a targeted risk reduction intervention through defined exercise (STRRIDE), long-term exercise interventions can be effective at improving and preventing further decline in, indices of skeletal muscle metabolic health, including whole-body insulin sensitivity, in sedentary, middle-aged adults with overweight and obesity [79, 80]. Concordantly, myostatin protein and plasma abundance were shown to be reduced in middle-aged insulin-resistant men, following long-term aerobic exercise, despite an absence of weight loss or a reduction in fat mass [17]. Thus, further research is necessary to delineate between the independent effects of advancing age, adiposity and physical inactivity in vivo [81]. Investigation to explore the possible involvement of other factors associated with the presence of excess adiposity in ageing, such as the ectopic accumulation of lipids in skeletal muscle and the role of physical activity/exercise levels in modulating myostatin expression, independent of adiposity, across the age-span, are therefore warranted.

## Limitations

Throughout the experiments reported herein, the expression of myostatin and of other factors associated with the regulation of skeletal muscle mass, was assessed only at the mRNA level. It must be considered, however, that changes in myostatin mRNA and protein expression do not typically correlate well, and their relative significance remains equivocal [82, 83]. Furthermore, studies of such association are limited by difficulties in reliably and reproducibly measuring muscle myostatin protein abundance due to differences in antibody specificities, varying interpretations of the numerous myostatin complexes/forms and discrepancies in their migration under different SDS-PAGE conditions [83].

The three groups in the cross-sectional analysis were generally well matched; however, ANA and AEA tended to present with less lean body mass than YNA. This difference is difficult to negate, since muscle mass almost universally declines with advanced age at a rate of ~1% per year, except in master athletes [84–87]. Whilst there is a limitation of this comparison, the modest loss of lean body mass in the aged groups is a common feature of ageing that reflects the general population. Furthermore, whilst myostatin mRNA expression was found to be significantly greater in AEA than ANA, the comparison between AEA and YNA did not reach statistical significance. It could therefore have been advantageous to have also recruited a young excess adiposity group to isolate the effects of ageing alone in the presence of excess adiposity and to have evaluated additional indices of the expression/regulation of myostatin and its signalling pathway, through the measurement of its muscle and circulating protein content and of the expression and activation of the SMAD 2/3 transcription factors.

Equal adipose tissue masses were used to generate the ACM used in this study. Therefore, whilst the model used effectively accounts for differences in the relative secretion of adipose-derived factors per unit mass, the effect of increased absolute adipose mass in individuals with excess adiposity was not captured. To that effect, in vitro studies using human serum from adults with differing levels of adiposity may also prove valuable. Furthermore, ACM was generated from donors who were undergoing total joint replacement due to OA. Thus, both groups likely presented with a more inflammatory phenotype than non-OA donors [88]. However, given the high rates of knee and hip replacement in aged adults and the presence of radiographic evidence of OA in > 50% of adults ≥ 65 years, the use of this patient group does not necessarily preclude comparisons with the general ageing population [89, 90].

Efforts were taken to create a physiologically relevant model of lipid-induced skeletal muscle insulin resistance. A combination of palmitic, linoleic and oleic acid was therefore applied to differentiated primary human myotubes, reflecting the three most abundant fatty acids within human plasma. The total fatty acid concentration in PLO (450 μM) was greater than that of palmitate alone (250 μM); however, this reflects the approximate combined abundance of these three free fatty acids within the human circulation and therefore allowed comparisons to be made with the isolated effects of physiological palmitate. Furthermore, it cannot be dismissed that different effects may occur in vivo in the setting of a chronic obesogenic diet, which warrants further investigation.

## Conclusions

Skeletal muscle myostatin mRNA expression is uniquely upregulated in aged adults with excess adiposity and insulin resistance but not by ageing alone. Indeed, the mRNA expression of myostatin was found to correlate with indices of adiposity but not age. Using in vitro models of human skeletal muscle, neither the secretory milieux of SAT derived from patients with excess adiposity, which did not induce insulin resistance but did perturb the expression of factors involved in the regulation of muscle mass, nor the overt induction of insulin resistance by elevated fatty acid availability replicated the upregulation of myostatin mRNA expression seen in vivo. Thus, the underlying factors responsible for the obesity-mediated upregulation of myostatin remain to be elucidated but appear not to be mediated by ageing or insulin resistance per se. Future research to identify such factors is warranted; given its prominence as a feature of excess adiposity in ageing, the role of intramuscular lipid accumulation should be examined.
